# Women and working in healthcare during the Covid-19 pandemic in Brazil: bullying of colleagues

**DOI:** 10.1186/s12992-023-00911-2

**Published:** 2023-02-18

**Authors:** Paulo Roberto da Silva, Paloma Porto, Mariela Campos Rocha, Eduardo Ryô Tamaki, Marcela Garcia Corrêa, Michelle Fernandez, Gabriela Lotta, Denise Nacif Pimenta

**Affiliations:** 1 Collective Health at René Rachou Institute – Fiocruz Minas, Belo Horizonte, Minas Gerais Brazil; 2 Social Anthropology at Federal University of Rio Grande do Sul (UFRGS), Porto Alegre, Brazil; 3 Political Science at the German Institute for Global and Area Studies, Hamburg, Germany; 4 Public Administration and Government from Getulio Vargas Foundation (FGV), São Paulo, Brazil; 5 Political Science Institute of the University of Brasília (IPOL/UnB), Brasília, Brazil

**Keywords:** Bullying, Covid-19, Caregiving, Gender, Women

## Abstract

**Background:**

Based on a feminist approach, we analyzed the experiences of workplace bullying suffered by women front-line healthcare professionals dealing with the Covid-19 pandemic. We start from studies that show that women make up 70% of the global health workforce, 85% in the area of nursing, and 90% in the case of social care workers. An unequivocal need thus exists to address gender issues regarding the composition of the labor force in the health area. The pandemic has aggravated recurring problems involving healthcare professionals at the various caregiving levels, such as mental harassment (bullying) and its effects on mental health.

**Methods:**

Data were gathered from an online survey of a convenience (non-probability) sample composed of 1,430 volunteer respondents, all women that work in the public health system in Brazil. The analyses and discussions involved the responses to a questionnaire containing 12 closed-ended questions and one open-ended question.

**Results:**

The results revealed a context of workplace bullying aggravated by precarious material, institutional and organizational conditions in the area of health services against the backdrop of the Covid-19 pandemic in Brazil. This context has variously led to aggression, isolation, heavy workloads, and invasion of privacy, humiliation, persecution and fear as it was possible to see, mainly, in the answers to the study’s open-ended question. This situation degrades both work relations and the integrity of the healthcare professionals who work on the front line to treat Covid-19 cases.

**Conclusion:**

We conclude that bullying is a psychosocial phenomenon that heightens the oppression and subordination still experienced by women in the contemporary context, but with new hues in a scenario of frontline response to Covid-19.

**Supplementary Information:**

The online version contains supplementary material available at 10.1186/s12992-023-00911-2.

## Background

The Covid-19 pandemic undoubtedly is the greatest humanitarian and scientific challenge faced by humanity since the end of the Second World War [1], demanding the adoption of a series of measures seeking to reduce contagion, such as wearing face mask, social distancing, and hand hygiene, that hamper the activities of a large portion of society. However, the subsequent social isolation and distancing measures do not apply to those deemed “essential”, among which are female healthcare professionals [2]. These professionals include a large and diversified set of “essential” people [2], i.e., healthcare practitioners on the front line of the response to Covid-19, to whom the dictum “stay at home” does not apply [2].

Women make up 70% of the global health workforce, 85% in the area of nursing [3], and 90% in the case of social care workers [4]. Women also represent more than 70% of the contamination cases by coronavirus of healthcare professionals in countries like the United States, Italy, and Spain [4]. An unequivocal need thus exists to address gender issues regarding the composition of the labor force in the health area, especially in personal caregiving [5] and exposure of women to contagion by Covid-19 [6].

Consequently, during the Covid-19, the daily routine of these healthcare workers has been strongly marked by feelings of fear and discrimination. The lack of access to personal protective equipment, lack of training, and exhausting work shifts amount to a series of ripple effects to which personal safety is just the tip of the iceberg. Long routines which lead to increased exposure to the virus, fear of transmission to family members, and discrimination - as they are often seen as possible agents of contamination - drives many to isolation. On top of that, confronted with the need to make decisions with a direct impact on the survival of patients, the mental health of many healthcare workers have been deeply impacted [2, 7–13].

To the scenario of more precarious employment, lower wages, more strenuous work shifts, and work-related accidents experienced by working women worldwide, it is necessary to add the problem of mental harassment at work or workplace bullying [14–17]. This bullying, which causes humiliation and suffering [17], is a worrying phenomenon in the contemporary world of work and has been called a veritable “Black Plague” of the twenty-first century [18].

In light of this, we present our research question: how have the places of oppression and subordination experienced by women today been reinforced in the context of the COVID-19 pandemic, when analyzing the experiences of moral harassment experienced by health professionals? We analyze the workplace experiences of bullying suffered by healthcare workers in the front line of the response to Covid-19 from a gender perspective, with a focus on women. Our aim is to understand how the oppression and subordination faced by women have been reinforced and intensified in the context of the pandemic, focusing on the analysis of reported situations of mental harassment.

We started from the assumption that the Covid-19 pandemic has been exacerbated by a systematic vulnerabilization of health systems over the past decades. The pandemic has intensified recurring problems involving healthcare professionals at varied caregiving levels, such as bullying and its adverse effects on mental health. For this purpose, we focus on the Brazilian case during the Covid-19 pandemic, bearing in mind the deterioration of healthcare systems resulting from reforms of social policies [19] instituted by political leaders.

In the Brazilian case, under the need to promote immediate responses to the challenges unleashed due the pandemic, the Federal Government has published the Temporary Providence (TP) 927/20 [20], which aims to slacken labor rules, in order to face the economics effects and preserve jobs and people’s income. The TP 927/20 effects in healthcare professionals were the suspension of administrative demands in security and welfare at work, the increase of the working day (including unhealthy activities), the suspension of vacation rights and non-paid licenses, as many others [21]. However, besides the hope for containing the effects of the pandemic, it is also considered the bother of keeping up the status quo of working relations, which is a mark of a long time context formed before the appearance of the new coronavirus, condition that confirms the harassing component at working context.

The overload of the health system raises quite a lot the risk of collapse in health services, increasing hence the damages to mental health of healthcare professionals, who already deal with situations such as physical and mental illness, psychology suffering and the worse of interpersonal working relations, especially when we make the division by genre in those experiences.

We begin with the assumption that the women who work in the front line of Covid-19 have experienced more situations of bullying as a reflection of working in a capitalist system that transforms the contradictions and constraints of the world of work into conflicts with strong subjective and interpersonal content [22]. As a multifaceted phenomenon that involves the expression of power and authority, mental harassment is present in the workplace in the form of abuse of power, verbal violence, physical violence, psychological violence, situations of systematic humiliation, persecution, isolation, and public embarrassment, producing interpersonal conflicts, and eroding the self-image of workers [22–25]. Bullying’s effects provoke severe consequences for physical health (general aches and pains and gastrointestinal, dermatological and cardiac problems), mental health (stress, fear, anxiety, apathy, low concentration, depression and suicidal thoughts), and organizational well-being (degradation of the working environment, demoralization of teams, low labor productivity, damage to equipment, high employee turnover and formal complaints), causing human, financial, and organizational losses [26]. Consequently, we assume that the reaction to workplace bullying should involve a critical and transformative stance to overcome mistreatment [27].

Concerning health workers, studies have revealed that mental harassment is a routine practice, especially acute among nurses, who are overwhelmingly women [25, 28–32]. We thus believe it is necessary to adopt an analytic lens that considers the consubstantiality of social relations in the world of work [33], to shed light on how specific groups are impacted differently by the phenomenon of bullying, as is the case of women and other minorities [34, 35].

## Methods

The data analysis was carried out by Getúlio Vargas Foundation, through a partnership between Oswaldo Cruz Foundation and Bill & Melinda Gates Foundation. This study was approved by the ethics committees of the respective institutions, as demonstrated by the Certificates of Presentation for Ethical Consideration (CAAEs): Research Ethics Committee of Federal University of Rio Grande do Sul (PROPESQ/UFRGS, CAAE 39832520.1.0000.5347); and Ethics Committee of the René Rachou Institute (Fiocruz Minas, CAAE 39832520.1.3001.5091).

It comes from an online survey that targeted healthcare professionals who work in the public health system during the Covid-19 pandemic in Brazil. The questionnaire was applied in March 2021, a moment of extreme pressure in Brazilian hospitals and health facilities. The country, at the time, was facing one of the worst waves of cases and deaths, and has reached more than 3,000 deaths per day.

The intention of this major study was to understand the impacts of the Covid-19 pandemic on healthcare professionals in various aspects: personal, social, psychological, and workplace relations. The data was collected by convenience, which means that our sampling procedure followed a subjective judgment rather than random selection. Therefore, we were limited to the voluntary responses of healthcare professionals, which does not allow us to make any statistical inferences about the population but limits our analysis to the sample of healthcare professionals interviewed. In total, there were 1,829 respondents, of which 1,430 were female - whose responses are the only considered in this article.

The difficulties imposed by the pandemic hampered the application of a probabilistic sampling design. Therefore, anchored on one of the criteria listed by Mattar [36], the urgent context of the pandemic allowed greater acceptability of using a nonprobabilistic convenience sample, due to the opportunity to fill a gap, namely the lack of summarized and descriptive statistics about the reality of these frontline professionals [36, 37].

The format of the questionnaire applied in the present investigation also finds support in studies conducted by other research groups in various countries examining the conditions of healthcare professionals in the fight against the new coronavirus [38, 39] and in the context of previous pandemics [40, 41].

The original questionnaire contained 52 questions of different natures, such as open-ended, binary and multiple-choice, etc. For the study discussed in this article, the analyses and discussions were carried out based on the responses of the 1,430 female healthcare professionals of the Brazilian public health system. The variables considered gathered of the responses to12 of the 52 questions. The ​​consent for the research was given at the beginning of the online questionnaire. The full questionnaire can be accessed in Supplementary Materials.

The quantitative analysis consisted of the calculation of descriptive statistics from the responses to the 12 closed-ended questions, addressing the following topics: receipt of the necessary equipment to face the coronavirus; receipt of training to deal with the pandemic; receipt of testing material; receipt of guidance, information and emotional support from supervisors during the pandemic; impacts on mental health; receipt of support to care for mental health problems and the persons in charge of this support; emotions felt in the contact with the public during the pandemic; the strategies employed to remain motivated and/or safe; the possible experience of mental harassment during the pandemic, and if so, the person responsible for this bullying.

In turn, the qualitative analysis involved content analysis of the response to the following open-ended question by the respondents who stated they had suffered some type of bullying during the pandemic: *“Would you like to share your story with us? (with assurance of anonymity and confidentiality).”*

In the analysis of the answers to the open question, the Content Analysis method was used [42], which made it possible to find regularities in the analyzed text. The method allows establishing groupings of elements of closer meanings, enabling the formation of more general categories of content. The construction of the categories followed the following path: (1) general reading and notes on the recurring elements in the answers; (2) counting the identified elements and establishing the general categories; (3) construction of the category grids containing: general theme, words and phrases linked to the theme; (4) grouping of words and phrases in the category and final counting of frequencies. Three categories of analysis were constructed and named as: Workplace bullying; Conflicts managed during work, and Degradation of the self.

The decision to focus only on women was based on two aspects. The first was the historic gender inequality and sexual division of labor criticized in the writings of female authors^1^[44, 45], showing that only a careful look at the markers of inequality can reveal the complexity of women’s demands. The second aspect refers to the fact that females make up some 70% of the health sector [46].

Moreover, the relevance of this study is based on three aspects. First, Brazil is one of the main epicenters of the Covid-19 pandemic in the world [47, 48]. Second, the country has a national public health system with a large female labor force that has been greatly affected by the pandemic. And third, Brazil suffers from high levels of inequality, including in the health labor force, making it relevant to understand the pandemic’s impact in this respect.

## Results

The results will be presented in quantitative and qualitative sections.

### Quantitative results

The online survey obtained valid responses from 1,829 people, 1,430 of them women (78.4%); it is worth highlighting that we obtained diversity in terms of race, with 47% self-declaring as white and 50.4% as black.

We also observe diversity in terms of age (7,3% 19–29 years, 31.1% 30–39 years, 36.2% 40–49 years, 21.1% 50–59 years, and 4.3% 60 and older); profession (9.8% doctors, 20,3% nurses, 49.6% community health agent-CHA/endemic response agent -ERA, and 20.3% others), and type of service (65.4% basic care, 8.8% specialized care, 17.2% hospital care, 0.7% management, and 7.9% other) for example. Nonetheless, there are also variables homogeneous distributions, such as sexual orientation, where 88.5% identify as heterosexual and 1.6% as homosexual. Table 1 below summarizes and illustrates the distribution of the other socio-demographic variables based on the sample of female respondents.

**Table 1:** Profile of the respondents

		Women
Total Respondents		1430(100)
		% (of Women)
Profession	Doctor	9.8%
	Nurse	20.3%
	CHA/ERA^a^	49.6%
	Other	20.3%
Service	Basic care	65.4%
	Specialized care	8.8%
	Hospital care	17.2%
	Management	0.7%
	Other	7.9%
Race	White	47.0%
	Black	50.4%
	Other	2.6%
Sexual orientation	Heterosexual	88.5%
	Homosexual	1.6%
	No response / Other^b^	9.9%
Afraid of the New Coronavirus	Yes, it is afraid of the new Coronavirus	11.5%
Readiness - Material and Organizational Conditions	Yes, feels prepared [to face the new Coronavirus]	27.8%
	No, it does not feels prepared [to face the new Coronavirus]	72.2%
Felt their mental health was negatively affected	Yes, they felt that their mental health had been negatively affected during the pandemic.	83.7%

Source: Survey (omitted). Note: 100% corresponds to the total number of respondents in each gender variable: (i) Women (*n* = 1430)

^a^*CHA *community health agent and *ERA *endemic response agent

^b^Other includes pansexuals and bisexuals

**Fig. 1:**
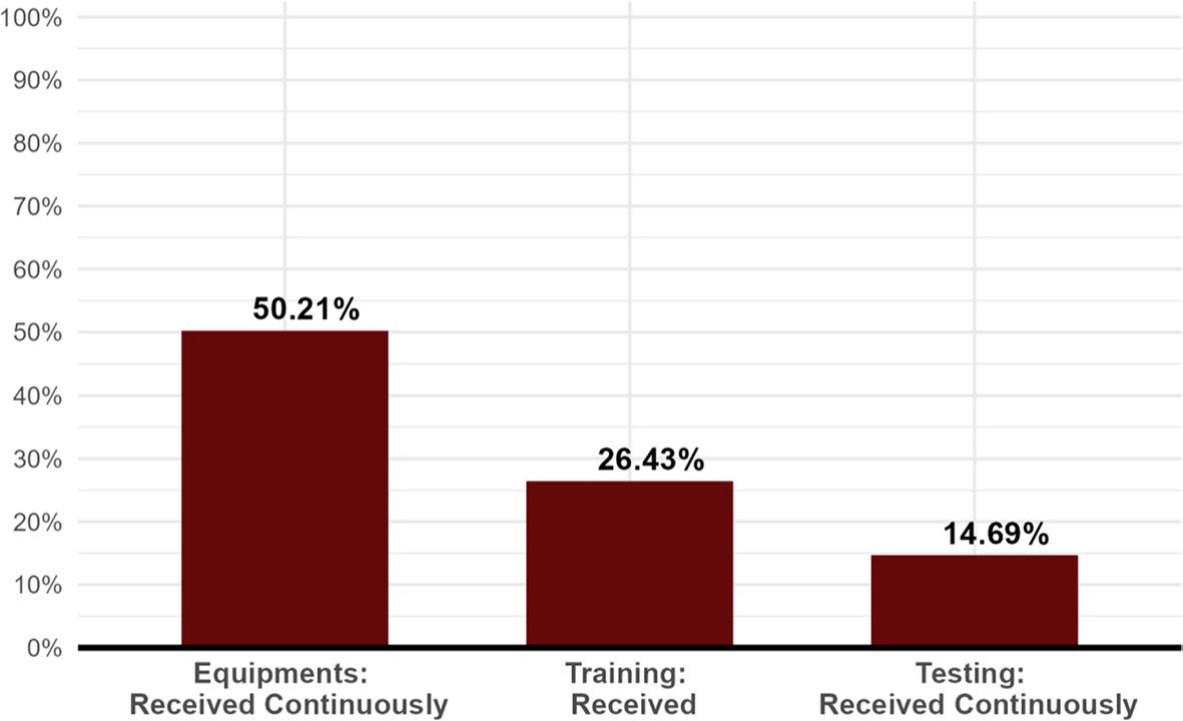
Receiving Equipments, Training, and Testing (%). Source: Survey (omitted). Note: 100% corresponds to the total number of respondents: (i) Women (*n* = 1430); The elements are not complementary. They are independent of each other. Each element can sum up 100% by itself

Concerning the material and organizational conditions related to work (Table 1), 27.8% of the respondents stated they felt prepared to face the coronavirus crisis. Not surprisingly, 88.5% felt afraid of contamination by the new virus (Table 1). When it came to receiving equipment, training, and testing (Fig. 1), 50.2% affirmed regularly receiving equipment, 26.4% just training, and only 14.7% continuous testing. Regarding support and orientation by superiors, 46.6% responded receiving emotional support from supervisors, and 61.6% received information about the pandemic from supervisors.

**Fig. 2:**
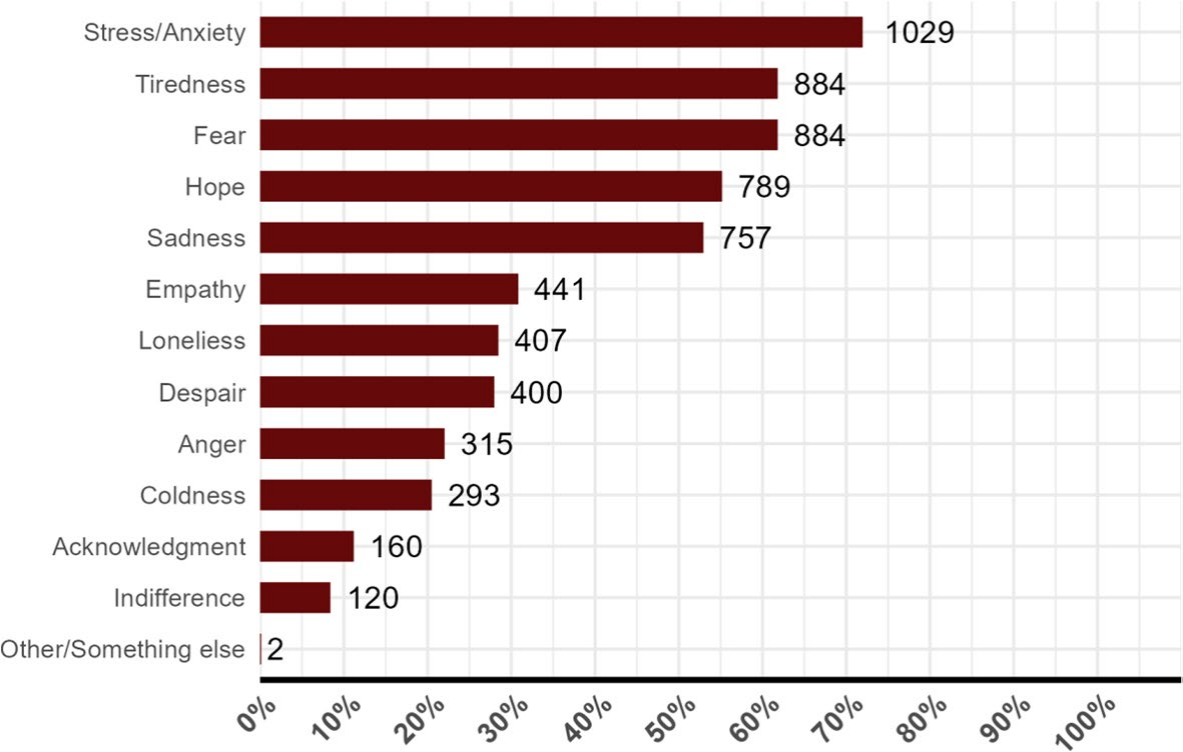
Personal mental health during the pandemic. Source: Survey (omitted). Note: 100% corresponds to the total number of respondents: (i) Women (*n* = 1430); The elements are not complementary. They are independent of each other. Each element can sum up 100% by itself

With regard to mental health (Table 1), 83.7% of the respondents stated that their mental health was negatively affected during the pandemic. The complaints were anxiety (71.9%), exhaustion (61.8%), fear (61.8%), sadness (52.9%), despair (27.9%), solitude (28.4%), and anger (22.0%), with the possibility of choosing more than one emotion (Fig. 2).

Finally, on the matter of bullying, of the valid responses, 34% of the healthcare professionals stated they had been victims. Table 2 present detailed information about the mental harassment experienced by the women.

**Table 2:** Role of the bully against female healthcare workers during the COVID-19 pandemic in Brazil

	n(%)	Increased during the pandemic (%)	Started during the pandemic (%)	The same as before the pandemic (%)
**Role of the bully**				
Supervisors	219 (100)	121 (55.25)	31 (14.15)	67 (30.6)
Users or clients	217 (100)	125 (57.6)	37 (17.05)	55 (25.35)
Colleagues (Female, male or any other gender? Or the information was not collected)	99 (100)	51 (51.52)	19 (19.19)	29 (29.29)
Head of State (is this the same as a politician?)	77 (100)	44 (57.14)	12 (15.59)	21 (27.27)
Relatives of users or clients	81 (100)	53 (65.43)	11 (13.58)	17 (20.99)
People in the streets or the general public	81 (100)	58 (71.61)	12 (14.81)	11 (13.58)
Others	6 (100)	5 (83.33)	1 (16.67)	0 (0)

Source: Survey (omitted). Note: 100% corresponds to the total number of respondents that suffered bullying: (i) Women who suffered bullying (*n* = 484)

### Qualitative results

The qualitative results were established from the open-ended question asked in the questionnaire: *“Would you like to share your story with us? (with assurance of anonymity and confidentiality).”* In relation to bullying, 209 female healthcare professionals narrated work situations in which they felt harassed. From the content analysis method three categories of analysis were built: Workplace bullying; Conflicts managed during work, and Degradation of the self.

Category: Workplace bullying.

We selected for this category of analysis the reports of the professionals related to context of distressing work, with situations such as lack of personal protective equipment (PPE), need to purchase their own PPE, deviation of job duties, lack of information and training, low salaries, excessive work demands, disrespect for working hours, forced transfer of sectors, denial of added pay for unhealthy working conditions, absence of assistance from supervisors, and fear of contamination by the coronavirus.

*When we went to demand personal protection equipment (PPE) from the secretary of the previous administration, we asked why there was no type of dialog with management, including about our labor rights, and the secretary at that time got angry and said that things don’t happen at our pleasure. It was mortifying, without mentioning another secretary who tried at all cost to belittle us. (Woman, Community Health Agent, Northeast Region, Brown, 39 years old, Heterosexual)*

*The supervisors don’t give us any training when some flows or routines are changed, and on the first opportunity arrogantly demand that we perform. (Woman, Nurse, South Region, White, 32 years old, Heterosexual).*

*I define bullying as the obligation to exercise functions for which I do not feel qualified or protected, without support to deal with adversities (such as suspension of schools and the risk of contamination of family members) and performance demands without considering these difficulties. (Woman, Doctor, Midwest Region, Brown, 42 years old, Heterosexual).*

*Yes, bullying by my immediate superior (name omitted), who I am suing, even including physical aggression and failure to properly distribute PPE. I requested reassignment because of this. (Woman, Community Health Agent, Northeast Region, Brown, 36 years old, Bisexual)*

*The Health Department requires working without PPE. This is very humiliating. In reality, it is not mental harassment, but rather humiliation. (Woman, Community Health Agent, Northeast Region, Black, 52 years old, no response on sexual orientation)*

*I have worked in the profession for 35 years, since completing my training to work in the public health system, and I have never faced a situation as difficult and challenging as this one. At the start of the pandemic, I did not receive PPE, under the argument that I did not have direct contact with patients. I tried to explain, in vain, that as a psychologist, I engage in conversations, with constant emission of respiratory droplets for long periods, especially when I work at the bedside of patients. I cannot work at a distance of 2 meters from the patient. This condition places me at risk, and also poses a risk to the patient. Initially, I had problems purchasing, out of my own pocket, PPE items due to shortages in the market. It was a major struggle, initially to guarantee the right to use my own material, and subsequently to receive it from the institution. (Woman, Psychologist, Southeast Region, White, 59 years old, Heterosexual)*

Category: Conflicts managed during work.

The healthcare workers in the context of Covid-19 reported harassment by users of the service, supervisors, colleagues and head of State. Therefore, in this category of analysis we selected some accounts that mention bullying in different ways: vertically descending (from superiors to subordinates), vertically ascending (from subordinates to superiors), or horizontal (between professionals of the same hierarchical level).

*“I’m a nurse and have repeatedly heard comments from users and family members that I was not qualified to administer care, that I was trying to pass myself off as a doctor, or that I was there to “bar” medical treatment. I’ve heard countless comments besmirching my work and my long years of study, insinuations and direct claims that I have insufficient study for guidance regarding COVID-19 and other diseases.” (Woman, Nurse, South Region, White, 27 years old, Bisexual)*

*“The supervisor, during a general meeting, asked everyone to consider the balance of occurrences in 2020 to plan for 2021. The realization of projects is suspended due to the pandemic, but I was used as an example, to say there should be reconsideration of my continuation, since I had not achieved any progress. Another colleague was also mentioned, and the supervisor ended by saying there would be no more meetings for feedback, that the biased eye and oblique or punctilious comments (by a colleague) during the working meetings are indicative of the return expected of the work.”(Woman, Psychologist, Southeast Region, White, 38 years old, Heterosexual)*

*“I was breastfeeding when the pandemic started and I worked in an unhealthy area, so I requested a transfer to an area that was not unhealthy. The supervisor rejected this, and to top it off, even asked when I would breast feed.”(Woman, Doctor, Southeast Region, White, 37 years old, Heterosexual)*

*“I left my management job with the Rio de Janeiro municipal government because I could no longer bear the harassment and mistreatment from the people of the previous government. I worked directly with Covid, health surveillance and primary care, and the work made both me and my colleagues sick.”(Woman, Service manager, Southeast Region, White, 29 years old, Heterosexual)*

Category: Degradation of the self.

We selected in this category the reports of the self-degradation and self-integrity of the healthcare professionals, from the subjective experience of sadness, depression, sleep disorders, anxiety, headaches, fear, solitude, symptoms of stress, professional denigration, fear of losing employment, emotional fever and sobbing.

*“The head nurse at the post where I worked treated me like a retard, laughed at me, because I was mentally tired, stressed out as the doctor put it, and she used this to ridicule me and was always very crass with me and the others, and my supervisor liked to say to my face that my colleagues did not like to pair up with me because I did not know how to use the computer and always needed guidance and she did not have the patience to explain things to me, and even said these things in the presence of patients.” (Woman, Community Health Agent, South Region, Brown, 42 years old, Heterosexual)*

*“I divide my working time between two group centers. At the start of the pandemic, the service continued to be open and I expressed the position in favor of temporary closing and adoption of remote work, based on the large risk run by everyone from in-person activities, both of workers and users. I was also very wary of everything, and like everyone, had to learn to deal with the new context. At one of the centers, I was heard and heeded. At the other, I was constantly harassed. I received harassing messages disguised as work messages (with orders and insinuations) in private. Even though I was part of a working group, I was often received with silence in response to my suggestions in the group. The manager personally told me, in the presence of my colleagues, that she had not read a message from me to spare her. I took my vacation on a different date than originally planned and for this I missed the first meeting after I returned to in-person activities in June 2020. The work mates who I got along with the best had their schedules changed so as not to work with me. Other colleagues assumed the same stance and started to treat me with indifference and even urged a close colleague of mine to avoid contact with me so as not to suffer reprisals from the manager. (Woman, Artist, Southeast Region, White, 37 years old, Heterosexual)*

*“For holding a public health management position, I suffered various verbal attacks in social networks due to municipal decrees restricting social movement. I had to lodge a formal complaint at a police station against one user for a racist epithet. I am the secretary of health of a city with low human development Index in the Cerrado region of Bahia. I got pregnant but suffered a miscarriage, had psychological problems, caught Covid, suffered respiratory aftereffects, but still continued to work. I had to take tranquilizers to sleep for several months. I still feel guilty about the miscarriage, because I correlated it with excessive work. I had insomnia, excessive weight gain, mood changes. I considered giving up my consulting work, because whenever I dedicated myself to it, something serious happened and I had to leave to take measures to record the deaths caused by Covid. (Woman, Service Manager, Northeast Region, Brown, 44 years old, Heterosexual)*

*“Better, no; I haven’t experienced a favorable moment when the possibility existed of my losing emotional control.” (Woman, Nurse, Southeast Region, Brown, 57 years old, Heterosexual)*

*“I don’t believe in confidentiality, anonymity. For this reason I prefer not to share anything. I have already suffered mental harassment from the mayor and her subordinates. I only want to do my work, to work in peace.”(Woman, Doctor, South Region, 44 years old, Heterosexual)*

## Discussion

These data allow us to point out that the daily experiences of the health professionals was strongly marked by moral harassment.The environment was characterized by precarisation and conflict that inflicted psychological consequences and even injury.

Mental harassment at work is defined as a series of intentionally abusive behaviors, repeated in time, to humiliate and socially exclude a person in the workplace. The consequences of bullying, such as self-degradation and weakening of identity, are increasingly reported by healthcare professionals, with nurses being the most affected [28, 49]. Within these teams, women are the primary victims of this type of psychosocial violence, given the feminization of healthcare professions [50]. It should also be notedthat the chain of command within hospitals is doctors, nurses, and nursing assistants/orderlies, with the last two categories being responsible for carrying out orders [51, 52].

In this regard, rigid and vertical hierarchical relations - often predominant in the majority of hospitals [53] - are favorable to the transformation of conflicts inherent to the work organization into situations of mental harassment, which results in psychosocial degradation of workers by making them doubt themselves as professionals, request transfer to other sectors, or resign. This rigidly hierarchical context in the health field is associated with reducing professional teams, material precariousness, intensifying work with long and exhaustive shifts, and the requirement for a polymorphous professional profile of multiple skills. The Covid-19 pandemic has greatly aggravated this situation.

Thus, this scenario configures what has been called “*distressing work*” [22] within the context of bullying. “*Distressing work*” is work that is impossible to complete adequately, or truncated work, against a backdrop of contradictions and constraints that materialize in organizations, making workers susceptible to bullying. The interviewees pointed out several experiences related to this: obligations that could not be fulfilled, lack of material conditions to carry out the tasks, mismatched information.

The new cycle of increased precariousness of working conditions in the area of health, associated with the overload of the health system and impending risk of collapse of health services in the context of Covid-19, has contributed to intensifying workplace bullying, as in the case of a group of 67 resident health multi-professionals (51 women and 16 men) of a large university hospital in the state of Rio Grande do Norte, of whom 44.8% reported suffering bullying during their residency [54]. In the nursing area, another case is emblematic by revealing exponential growth of the phenomenon among female professionals in the context of the Covid-19 pandemic, with the significant presence of situations of physical, verbal, and psychological aggression [55].

The reports of our respondents depicted a wider context of distressing work, with situations such as lack of personal protective equipment (PPE), need to purchase their own PPE, deviation of job duties, lack of information and training, low salaries, excessive work demands, disrespect for working hours, forced transfer of sectors, denial of added pay for unhealthy working conditions, absence of assistance from supervisors, and fear of contamination by the coronavirus.

As a manifestation of distressing work caused by the exploitation of the workforce in the capitalist context, such as long and exhausting work shifts, reduction of salaries, diminished worker protection, greater precariousness, imposition of unreasonable goals, increase of informal labor, growing extraction of surplus value and individualization of problems [25], bullying requires a level of comprehension that places the contemporary capitalist mode of production and its socio-metabolic effects as central elements, thus displacing analyses seeking individual responses and even leading to solutions through judicialization of the phenomenon. That analysis also requires understanding that the conflicts managed in the workplace become personal dilemmas and experiences of suffering and humiliation. Health professionals therefore took upon themselves a responsibility, an impossible workload, which they should not have, and this produced an excessive burden on them.

Studies of healthcare teams have shown that excessive delegation of activities, supply of confusing and imprecise information, failure to convey information useful for work, giving instructions impossible to carry out, depriving workers of access to work instruments, inducing errors, assigning incompatible tasks, imposing unjustifiable working hours, restricting labor rights such as vacation, and salary discrimination [49] are situations of bullying reported by healthcare professionals, mostly women, in their working routine. This description is very close to that depicted by the quantitative and qualitative data gathered by our survey.

The work of caregiving, as we have argued, involves contact between bodies, relations of proximity and subjective implication of healthcare workers, requiring them to experience moral dilemmas in the context of Covid-19 that involve their own safety and that of others. Health professionals felt responsible for the life and death of patients, something that should be a responsibility of the health service.

The invitation to the accountability of their actions regarding the lives of others [16] thus has become a situation present in the everyday routine of these healthcare professionals in the context of Covid-19, causing transformation of the material and organizational adversities, i.e., distressing work, into psychosocial conflicts, strengthening the subordination and oppression that women experience, outside and within the context of healthcare work. In this sense, we believe that the mental harassment, experienced as psychosocial in nature, is a phenomenon resulting from a distressing and precarious work environment, which demands a degree of knowledge for analysis that encompasses a restructuring of capitalism and its effects on the subjectivity of social relations, especially the effects on women, producing constraints, humiliation, embarrassment or even desire to abandon work, as indicated in the narratives. Thus, women are the main victims of a *modus operandi* of organizing and managing work, intensified in the context of Covid-19, which has transformed the working environment into a minefield marked by fear, disputes and abuses of power, causing suffering and physical and mental overload of workers [17].

Work in the health area is performed most of the time by multi-professional teams, with rigid vertical organization, carried out under pressure and subject to multiple conflicts arising from the interpersonal relations with workmates, patients and their family members [54]. Therefore, activity in the healthcare area is fertile terrain for transformation of disputes into situations of violence and violation of rights, characterizing bullying, especially in the context of a pandemic in which the novelty of the virus and the risk of death make the working conditions even more precarious. The upshot is a context in which mental harassment passes through processes that are at the same time naturalized and reproduced by healthcare professionals, without critical analysis of the harmful effects.

Against this backdrop of work under pressure, bullying can occur through different routes: vertically descending (from superiors to subordinates), vertically ascending (from subordinates to superiors), or horizontal (between professionals of the same hierarchical level) [51]. As seen in the quantitative data, the healthcare workers in the context of Covid-19 reported harassment by users of the service, supervisors, colleagues and head of State. The health professionals’ reports show the harassment experienced in their relationships with superiors, co-workers, patients and patients’ families, which demanded responsibilities that went beyond the individual sphere.

We have depicted some of the conflicts generated by the routine of the respondents that have put them at jeopardy of suffering gender violence, as in the case of questioning the right to breastfeeding. We consider these to be clear examples of how the Covid-19 context has aggravated the violence routinely suffered by women in the workplace, with mental harassment acting as a springboard of this oppression. Various other situations of perturbation and humiliation were mentioned, such as attacks for becoming sick, accusation of stealing vaccines, accusation of contaminating patients or work mates, doubt regarding pregnancy, demands to do the work of colleagues who do not want to become infected, and reassignment of the work location as punishment. Whether vertical or horizontal, from users, colleagues, supervisors or head of State, what matters is not only to understand the perverse nature in the figure of the aggressors as an explanation for the phenomenon of bullying experienced by healthcare professionals in the context of Covid-19, but also to find in the organization of the world of work, in the processes of restructuring health services, and in the securitization and feminization of health services, the central elements that transform interpersonal relations into relations of violence and denial of others [51], especially violence against women.

It is important to stress that the work of caregiving, or caregiving as work, is the expression of the sexual division of work produced by the capitalist mode of production, which exploits women and does not separate the reproductive from the productive sphere [56–59]. Therefore, historically men have occupied social positions in the productive sphere, with remunerated work, while women have been assigned to roles in the reproductive sphere, with unpaid tasks, and when they enter the labor market, they are impelled to reproduce the caregiving, based on the symbolism of the maternal metaphor of dedication, abnegation, sacrifice, submission and natural love for the caregiving mission [60]. We consider it important to emphasise that these same demands are never placed on male professionals.

It is not without reason that the two agents of bullying identified the most by the healthcare professionals were supervisors and users. The supervisor (sometimes a physician) is at the top of the line of command of health services, and the users are those who demand that the body, devotion, subjectivity, emotion and negotiation appear in the scene, leading to situations in which the professionals are psychologically exhausted and doubt themselves in the exercise of their profession, as a clear expression of the degradation of their integrity and subjectivity.

Starting from comprehension of bullying as a reflection of sociability based on capital, of a contemporary restructuring of the world of work that produces situations of interpersonal conflicts and professional traumas that are manifested in behaviors such as threats of firing, persecution, strict disciplinary control, insults, accusations and verbal aggression, which gravely injure the subjectivity and mental health of workers [22], we analyzed how these situations produce consequences that affect the self-degradation and self-integrity [61] of the healthcare professionals.

A study of caregiving work [62] showed how the routine of these workers has been invaded by demands expressed by those they care for, the use of the body, emotions, devotion and decisions, causing these healthcare workers, especially in the context of the pandemic, to experience anxiety, depression, anguish, sleep disorders, use of drugs, fear, burnout syndrome, post-traumatic stress disorder (PTSD), negative social behaviors and mental exhaustion, as part of the impacts caused to their mental health.

In the case of the healthcare professionals who took part in this study, the scenario is similar. Their narratives identified painful experiences caused by situations such as: inappropriate criticism, isolation, humiliation, denigration, demands outside work, ridicule, harassment or undue public exposure, threatened dismissal, verbal aggression, physical aggression, threatened dismissal, disregard of opinions, investigation of personal life, persecution, defamation and exposure in work-related message groups.

Situations like these experienced by healthcare professionals were also described in a survey of 259 nurses working at basic health clinics and public hospitals [49] communication by shouting; aggression in private; invasion of private life with phone calls, e-mails and letters; physical aggression; refusal to listen in the presence of others; interruption of comments; threats of transfer to other sectors for the purpose of isolation; communication only in writing; and prohibition of communication by colleagues.

With regard to the subjective experiences, we stress that the healthcare professionals surveyed described sadness, depression, sleep disorders, anxiety, headaches, fear, solitude, symptoms of stress, professional denigration, fear of losing employment, emotional fever and sobbing. These are consequences of mental harassment that cannot be seen as natural phenomena inherent to working conditions. This would make their comprehension more difficult, since naturalization creates a smokescreen that obfuscates the real causes of the phenomena and fails to show their effects, shifting the focus to addressing consequences instead of the causes [25, 28–31].

Self-degradation thus leads to degradation of physical and mental health of the victims, lack of their possibility of acting, and a situation of confrontation in their personal and professional identities, at the extreme causing them to feel guilty for the situation, as can be seen in the accounts presented. Guilt therefore functions as a mechanism that transfers responsibility and produces damaging psychological effects on these professionals.

The self-blame, anxiety and discomfort experienced are signs of this degradation of mental functioning and psycho-affective balance [63] experienced by the healthcare professionals. This can be blamed on the discredit in the eyes of supervisors, colleagues and users of services; accusations of incompetence; manipulation of colleagues; hindrances to professional growth; humiliation and embarrassment in public, forced transfer to other sectors and isolation at work. The bullying manifested by these situations generates consequences, such as alterations in the capacity for concentration and judgment, sadness, depression, suicidal thoughts and use of psychoactive substances [50], besides stigmatization in the workplace by means of descriptions such as “overly sensitive”, “paranoid” or “victimist” [49].

With the victim discredited in the workplace, she is discouraged from sharing her experiences with others, preferring silence, since she is afraid of losing her job or of being transferred to another sector. Because many companies and institutions do not have institutional ways to resolve situations like this, each victim is harassed by her own suffering [51]. This leads us to question whether these might have been elements prompting so many respondents in our study not to recount their experiences in the open-ended question.

What can be perceived from these narratives is that the experiences of workplace bullying have become unsettling, so people often avoid speaking about them, since this can bring unpleasant memories, thoughts or images of the aggressions suffered [51]. This is a psychosocial phenomenon that produces a type of subjective crisis, a level of mental debility that degrades work relations and the victim herself. And who cares for the caregivers? If a pact of tolerance and silence exists in reaction to situations experienced in the context of work, on whom can the victim rely for help? For this reason, it is necessary to devolve this question to the organizations and seek responses that aim both to denaturalize and make visible the consequences of mental harassment to the victims, organization and society.

## Conclusion

The Brazilian society has been intensively affected over the last decades by reforms at the working market, which provoked a systematic vulnerabilization in health systems [16]. Such vulnerabilization is highlighted by the increase of outsourcing jobs, as well as of temporary working contracts, of labor rights’ loss, of working intensification, of most precarious working conditions, the reduction of wages and the spread of physical and mental suffering with regard to the job [64]. Such context has been aggravated by Covid-19 pandemic, showing clearly the negative effects of those reforms.

Literature reviews [25, 65] have indicated the relative lack of studies examining the gender and race dimensions in the manifestation, comprehension and resolution of workplace harassment. Based on the analyses presented here, we stress that another theoretical-methodological gap exists in the empirical evidence of the different impacts of this phenomenon on these groups.

We have sought in this article to analyze the types of mental harassment experienced by a sample of healthcare professionals involved in the frontline response to the Covid-19 pandemic in Brazil. We stress that in this context, mental harassment is a psychosocial phenomenon that ratifies the oppression and subordination experienced by women in contemporary society. The analyses revealed a context of distressing work, based on precarious material, institutional and organizational working conditions, placing the healthcare professionals in various types of conflicts: with supervisors, colleagues and users of health services. The caregiving work requires these professionals to mobilize their bodies, subjective feelings, emotions and senses of responsibility while attending patients, in a pandemic context of hindrances and contradictions. This context thus produces aggressions, isolation, exaggerated work demands, invasion of privacy, humiliation, persecution and fear, in turn leading both to degradation of work relations and degradation of self-integrity.

Even if the focus of our text is related to the experiences of moral harassment experienced by healthcare professionals that acted into the context of Covid-19, we consider important to highlight that those women related situations of care among themselves, of appreciation and recognition due the work accomplished by population and leaderships, of reception of the suffering from pairs, and of construction of different memories. It is important to understand the relationship between the mental condition of frontline workers and their performance during the policy implementation, especially in times of crisis. In this way, experiences of moral harassment can directly impact the quality of the health service offered to the population.

Therefore, it is important to think about interventions in the context of work to help face mental harassment as a phenomenon underpinned by global capitalism, such as: 1 – encouraging the conduction of studies about the different manifestations of bullying in the area of health, in the sense of denaturalizing it and making it increasingly visible; 2 – creating institutional policies for lodging complaints, investigating them and offering psychosocial support to victims; and 3 – creating educational programs within organizations with reviews of management practices, training of leaders and management of conflicts.

The proposal of these strategies is thus based on an anti-psychological and anti-legalistic interpretation of mental harassment, because one cannot expect to find its causes in individual aspects, but rather in the capitalist management of work. We do not propose a policy of response involving judicialization of cases, but instead a revision of work management policies, so as to create conditions for dialog, realization, dignity and integrity of the exercise of professions, without reproducing violence and violations that reinforce oppression and subordination.

## Supplementary Information

**Additional file 1.**

## Data Availability

Both qualitative and quantitative data was available upon reasonable request by contacting Paulo Roberto da Silva Júnior (paulosilva.junior@yahoo.com.br).

## Abbreviations

CHA: community health agent
Covid19: Coronavirus Disease 2019
ERA: endemic response agent
PPE: personal protective equipment
TP: Temporary Providence

## Footnotes

1 The feminist eye is legitimate as a critique underpinning the fight against gender inequality stretching back more than 200 years. The struggle for gender equality, with roots in the French Revolution, became a topic of feminist literature starting in the nineteenth century and has gone through various interpretive waves since then. An important milestone was the publication in 1792 of a manifesto on women’s rights written by Mary Wollstonecraft [43], “but that is still not legitimized/known among many women and is strongly opposed by patriarchy”.
