# Supplementary material for: Women and working in healthcare during the Covid-19 pandemic in Brazil: bullying of colleagues
Source: Global Health. 2023 Feb 18;19:10. doi: 10.1186/s12992-023-00911-2 (PMC9938680; doi:10.1186/s12992-023-00911-2)
Supplement: Supplementary file 1 — Additional file 1. [file 12992_2023_911_MOESM1_ESM.docx]

**Quiz - Covid-19 and the Public Health Professionals**

1. Are you afraid of Covid-19 [Required; Binary]

[ ] Yes

[ ] No

1. Do you feel prepared to deal with Covid-19 crisis? [Required; Binary]

[ ] Yes

[ ] No

1. [If you answered ‘yes’ to the previous question] What else contributed so that you fell this way? [Optional]
2. Did you receive the equipment needed to face the new Coronavirus? [Required; single choice]

[ ] I received continuously during the crisis

[ ] I received once or a few times during the crisis

[ ] I have never received

1. [If you answered ‘yes’ to the previous question] If you have received the equipment to deal with the pandemic, how do you rate its quality? [Required; Single Choice]

[ ] Great

[ ] Very good

[ ] Good

1. In case you have received the equipment to deal with the pandemic, how would you rate its quality? [Required; Single Choice]

[ ] Great

[ ] Very good

[ ] Good

[ ] Low quality

[ ] Terrible

1. Were you trained to deal with the Covid-19 crisis? [Required; binary]

[ ] Yes

[ ] No

1. Did the Covid-19 crisis alter the way you connect with the citizens? [Required; binary]

[ ] Yes

[ ] No

1. [If you answered ‘yes’ to the previous question] What has changed? [Optional]
2. Has the Covid crisis changed your work procedure? [Required; Binary]

[ ] Yes

[ ] No

1. [If you answered ‘yes’ to the previous question] What has changed? [Optional]
2. Do you know any co-workers who have COVID-19 or have any suspicions? [Required; Binary]

[ ] Yes

[ ] No

1. Who do you think are the most vulnerable users (of the service in which you operate) at this time of crisis? [Required; Multiple choice]

[ ] Seniors / elderly population

[ ] People with comorbidities

[ ] Women, pregnant or lactating

[ ] Children

[ ] Homeless population

[ ] People in poverty or extreme poverty

[ ] Black population

[ ] Other: ___

1. Did you receive testing material from the service you work for? [Required; Single Choice]

[ ] I received continuously during the crisis

[ ] Received once or a few times during the crisis

[ ] I never received

1. Have you received guidance from your boss on how to act during the crisis? [Required; binary]

[ ] Yes

[ ] No

1. Have you received support from your superiors to face the crisis? [Required; binary]

[ ] Yes

[ ] No

1. Do you believe the Federal Government has acted in a way to protect you? [Required; binary]

[ ] Yes

[ ] No

1. Do you believe the State Government has acted in a way to protect you? [Required; binary]

[ ] Yes

[ ] No

1. Do you believe the Municipal Government has acted in a way to protect you? [Required; binary]

[ ] Yes

[ ] No

1. A patient diagnosed with COVID19 requests a treatment that is not consensual in the science community, but much talked about on the internet. In your opinion, in this situation, professionals: [Required; Single Choice]

a) must give access to treatment. The user has the right to choose.

b) must not give access to treatment. The final word must belong to the health professionals

1. During the pandemic, medicines developed for other diseases have been used, as there is still no specific treatment for COVID19. Do you think this strategy: [Required; Single Choice]

a) must be used even if there is no evidence of the effectiveness of these drugs since we must do everything possible for the wellbeing of the user

b) Only drugs with proven efficacy should be used

1. In which service do you work? [Required; Single Choice]

[ ] Basic Attention

[ ] Specialized Attention

[ ] Hospital Attention

[ ] Other: ______

1. What is your profession? [Required; Single Choice]

[ ] Nursing Professional

[ ] Doctor

[ ] Psychologist

[ ] Physiotherapist

[ ] Endemic Combat Agent

[ ] Community Health Agent

[ ] Nutritionist

[ ] Services Manager

[ ] Other

1. How long have you been acting in this area? [Required; Single Choice]

[ ] Less than 5 years

[ ] Between 5 and 10 years

[ ] Between 10 and 15 years

[ ] Between 15 and 20 years

[ ] More than 20 years

1. Do you work in another health service? [Required; Single Choice]

[ ] Yes

[ ] No

1. [If you answered ‘yes’ to the previous question] In which one? [Required; Single Choice]

[ ] Basic Attention

[ ] Specialized Attention

[ ] Hospital Attention

[ ] Other: ______

1. What type of employment relationship do you have [Required; Single Choice]

[ ] Consolidation of Labor Laws

[ ] Position in tender

[ ] Statutory regime (public tender)

[ ] Outsourced

[ ] Collaborator (legal person, Individual Micro entrepreneur etc.)

[ ] Other: _____

1. Have you been coordinating with other services during the crisis? (such as schools, social care service centers, other health services, etc.) [Required; binary]

[ ] Yes

[ ] No

1. [If you answered ‘yes’ to the previous question] Which services have you been working on at the moment? [Required; Multiple choice]

[ ] Basic Attention

[ ] Specialized Attention

[ ] Hospital Attention

[ ] Social Care Service Centers (CRAS)

[ ] Specialized Reference Center for Social Assistance (CREAS)

[ ] Public Security (Military Police, Civil Guard, etc.)

[ ] Other: ____

1. In which Federation Unity do you work? [Required; Suspended Menu]
2. In which city/county do you work? [Optional]
3. How long have you been acting in this region? [Required; Single Choice]

[ ] Less than 5 years

[ ] Between 5 and 10 years

[ ] Between 10 and 15 years

[ ] Between 15 and 20 years

[ ] More than 20 years

1. Do you have any kind of connection with the territory in which you operate? [Required; Binary]

[ ] Yes

[ ] No

1. [If you answered ‘yes’ to the previous question] What kind of tie do you have with the territory? [Required; Multiple Choice]

[ ] The place where I was born

[ ] Current residence

[ ] I live in a nearby region

[ ] Family relationships

[ ] I attend the church/afro religion /worship services in the neighborhood

[ ] I attend leisure spaces

[ ] Affective/friendship relationships

[ ] Other: _____

1. How do you picture your work in the coming months? [Optional]
2. Do you believe your mental health was affected by the pandemics? [Required; Binary]

[ ] Yes

[ ] No

1. Have you received any support to take care of your mental health [Required; Binary]

[ ] Yes

[ ] No

1. [If you answered ‘yes’ to the previous question] What kind of support? [Optional]
2. Who do you turn to when you have problems with your mental health? [Required; Multiple choice]

[ ] Mental health professionals (psychologist, psychiatrist, etc.)

[ ] Relatives

[ ] Religious guide (priest/pastor/Mother or Father of Saint, etc.)

[ ] Friends

[ ] Coworkers

[ ] Boss/supervisor

[ ] Nobody

[ ] Other: ____

1. Which emotions did you feel in contact with citizens during the pandemic? [Required; Multiple choice]

[ ] Fear

[ ] Empathy

[ ] Rage

[ ] Pity

[ ] Affection/kindness

[ ] Distance/Coldness

[ ] Indifference

[ ] Mistrust

[ ] Other: ___

1. In your point of view, what situations caused these feelings? [Required; Multiple choice]

[ ] Social isolation

[ ] Social distancing

[ ] Interruption in face-to-face meetings

[ ] Health risk

[ ] Lack of physical contact

[ ] Interruption of work

[ ] Lack of PPE

[ ] Risk to the health of your family members

[ ] Other: ___

1. Which personal emotions did you feel / do you feel during the pandemics? [Required; Multiple Choice]

[ ] Fear

[ ] Empathy

[ ] Rage

[ ] Affection/kindness

[ ] Distance/Coldness

[ ] Stress/Anxiety

[ ] Loneliness

[ ] Hope

[ ] Tiredness

[ ] Hopelessness

[ ] Sadness

[ ] Indifference

[ ] Other: ___

1. In your point of view, what situations caused these feelings? [Required; Multiple choice]

[ ] Social isolation

[ ] Social distancing

[ ] Fall in family income

[ ] Interruption in face-to-face meetings

[ ] Possibility of getting infected

[ ] Uncertainty about the future

[ ] Interruption of work

[ ] Lack of PPE

[ ] Work overload

[ ] Risk to the health of your family members

[ ] Progressive increase in the spread of the virus

[ ] Lack of government action

[ ] Colleagues who have been diagnosed with Coronavirus

[ ] Psychological exhaustion

[ ] Physical exhaustion

[ ] See my colleagues scared

[ ] Lack of resources (investments, hospital beds, etc.)

[ ] Other: ___

1. What strategies have you adopted to keep yourself motivated and/or safe? [Required; Multiple Choice]

[ ] Humor and empathy with my colleagues

[ ] Solidarity

[ ] Purchase of personal PPE

[ ] Being close to family

[ ] Social isolation when not working

[ ] I don't feel motivated and/or safe

[ ] Other:_____

45. How do you feel about the growing process of reopening (bars, restaurants, malls and public spaces, for example) that has taken place in recent months? [Required; Single Choice] [ ] I am against reopening

[ ] I am in favor of reopening only essential services and with the use of a protective mask

[ ] I am in favor of a full reopening with the use of protective masks

[ ] I am in favor of a total reopening without mandatory mask use

1. Did you suffer any kind of moral harassment during the pandemics? [Required; Single choice]

[ ] Yes, and it was increased in the pandemics context

[ ] Yes, but it is the same as before

[ ] Yes, and it started with the pandemics

[ ] No

1. Would you like to share your story with us? (Reminding that we assure anonymity and confidentiality of the report) [Non mandatory; open]
2. Below are questions about topics related to scientific knowledge and I would like you to say whether you agree or disagree with these sentences [Optional]:

|  | Completely disagree | Partially disagree | Do not agree or disagree | Partially agree | Completely agree |
| --- | --- | --- | --- | --- | --- |
| The President can prescribe drugs for the treatment of COVID-19 to the population. |  |  |  |  |  |
| Staying at home, going outside as little as possible and always wearing a mask, and always washing your hands with soap are the most effective measures against COVID-19. |  |  |  |  |  |
| When it comes to my life and my health, I really believe in science. |  |  |  |  |  |
| Global warming ended 1998. |  |  |  |  |  |
| The human and social sciences are not, in fact, scientific. |  |  |  |  |  |

**Profile**

1. Gender Identity [Required; Single Choice]

[ ] Cisgender Woman*

[ ] Cisgender Man*

[ ] Transgender Woman

[ ] Transgender Man

[ ] Non-binary

[ ] Transvestite

[ ] I would rather not to say

[ ] Other: ____

*In gender studies, cisgenderness is the condition of a person whose gender identity matches the gender assigned to them at birth. For example, someone who identifies as female and was assigned female at birth is a cisgender female. The term cis is opposite to trans.

1. Race / Skin color [Required; Single choice]

[ ] Asian

[ ] White/caucasian

[ ] Indigenous

[ ] Brown

[ ] Black

[ ] I rather not to declare

1. Age [Required; Number]
2. In case you’d like to receive the final survey report, please leave your email or WhatsApp number (reminding that at no time will any respondent be identified) [Optional; open]
